# Nutritional supplements use, cost, source of information, and practices by Johannesburg North gym goers

**DOI:** 10.17159/2078-516X/2020/v32i1a6771

**Published:** 2020-01-01

**Authors:** Y Coopoo, X McCreanor, G Gabriels

**Affiliations:** 1 Department of Sport and Movement Studies, Faculty of Health Science, University of Johannesburg, Gauteng, South Africa; 2 Department of Pharmacy and Pharmacology, Faculty of Health Sciences, University of the Witwatersrand, Johannesburg, South Africa

**Keywords:** alternative protein, body image, cost, gender, spiritual motives

## Abstract

**Background:**

Nutritional supplements are defined as any dietary supplement manufactured product that is generally intended to supplement the diet when taken by mouth as a pill, capsule, tablet, or liquid. Currently, the use of nutritional supplements is on the increase worldwide, predominantly in Western countries but also more recently expanding to other parts of the world for what has become a multibillion-dollar global industry. As a result, consumer demand has caused the increase in the advertising and marketing of these products. This contributes to early exposure to nutritional supplements by potential consumers and is an influencing factor for the use of performance-enhancing and/or appearance substances by adolescents. For the nutritional supplement industry the container is thus the manifestation of innovative ideas for the enterprising business-minded mogul. For the consumer, body image and ideal body discrepancy, and social influences manifest in the belief that the perfection of body development cannot be achieved without the use of nutritional supplements. This makes the consumer a captive audience for the industry and a challenge for the health care provider when suggesting alternatives to nutritional supplements, based on cost-benefit, and risk assessment.

**Objective:**

To determine the association between commercial gym goers and nutritional supplements, in particular the commencement of use, reasons and purpose for use, and the financial and risk implications of use.

**Methods:**

A self-administered questionnaire based on a cross-sectional quantitative design and systematic convenience sampling was given to the 364 recruited males and female gym goers.

**Results:**

This study’s finding shows that the main reasons why females attend gyms are for muscle gain (57%), weight loss (48%), staying healthy (47%), and a ‘spiritual motive’ (39%) In males, it is predominantly for muscle gain (54%). Protein supplements were the most popular products that were consumed (84%) followed by carbohydrates (72%) and vitamins (71%). It was found that the consumption of nutritional supplements often starts at high school age and continues into adulthood. The analysis shows that natural source protein products are better priced than nutritional supplement products.

**Conclusion:**

The study shows the importance of educating gym goers, the general public, and the guardians of minors to make a behaviour change towards nutritional supplement consumption. The change should also incorporate a cost-benefit risk assessment which is practical for the consumer when comparing supplement use as alternative sources of protein.

In the area of nutritional supplements in general, the earlier work of Muller and Krawinkel indicates the extent of research that has been undertaken in the area of nutritional supplements.^[1]^ Nutritional supplements refer to any product that is generally intended to supplement the diet when taken by mouth as a pill, capsule, tablet, or liquid. The specific definition and categorisation is often grey, thus leading to definition complexity.^[2]^ Currently, the use of nutritional supplements is on the increase worldwide, predominantly in Western countries, but is also expanding to other parts of the world, including South Africa. ^[1]^ This is substantiated by the nutritional supplement sales figures for what now is a multibillion dollar global industry ^[2,3,4]^ projected in 2015 to be US$ 93.15 billion.^[3]^

The work of Starr and others provides evidence that the exposure to nutritional supplements in other parts of the world has been via advertisements, including print and Internet advertisements, and infomercials. ^[5]^ This pattern is also found in South Africa. Dodge and Jaccard provide links to the various forms of advertising ^[5]^ marketing and use of nutritional supplements, showing that exposure at an early age, such as, by adolescents ^[6]^, are factors that influence a behavioural intention for steroid users.^[5,6]^ The use of performance-enhancing and/or appearance substances in young adulthood. ^[2,5,7]^ is often a gateway to the use of more harmful and illicit substances, to achieve desired outcomes. ^[7]^ Recent work has also shown that the marketing practices of supplements is often dishonest and that many of the claimed benefits have little or no scientific supporting evidence ^[7]^ of product safety and efficacy. Approval from regulatory bodies has often not been obtained before marketing the supplements. ^[2]^

Further, unless the consumer has been found to have a nutrient deficiency, nutritional supplementation consumption may not improve the deficiency but may rather have detrimental effects on the consumer’s health. ^[2]^. In competitive sport, factors such as nutritional supplement consumption, can be a contributory factor in the determination of the results of sporting contests ^[2]^. For the gym user, particularly with endurance exercise training, nutrition plays an important role with regard to performance. ^[8]^ In the work by Morrison et al. that surveyed persons who exercised regularly at a gym, they showed that a majority of the gym users (85%) took supplements for the purpose that the product is intended and not for any potentially prohibited substances that may be in the supplements. Many consumed multivitamins (45%), protein shakes (42%), Vitamin C (35%) and Vitamin E (23%) at least five times per week. ^[9]^ According to Fitzgibbon et al. teenagers’ body image discrepancy and social influences were among the reasons for the use of supplements. ^[10]^ The use of supplements by female users was based on body dissatisfaction and the aspiration to be thinner. On the other hand, male users indicated that they required the use of supplements in order to develop their muscles. ^[11]^ Therefore the use of these supplements and their intended value may have shifted in the context of the gym goers’ desires and aspirations by today’s standards. This may indicate that for gym users the ideal body is not achievable unless they use nutritional supplements. ^[10]^ The impact of this shift is considered to be a potential health risk to the user due to the non-declaration of all ingredients on the product’s label and the associated lack of warnings and side effects included with product information. ^[12]^

In order to provide the gym user with an alternative approach to supplement use ^[7]^, the study uses protein requirements to illustrate the cost-benefit and risk assessment of a product. The aim of this study was therefore to assess, in the Johannesburg North region of South Africa, gym goers’ reasons for going to the gym, the specific nutritional products consumed by these users, and the monthly cost to attain their personal goals and needs.

## Methods

### Participants and study design

The study was conducted in the Johannesburg North region of South Africa at five selected and reputable gyms. The recruitment for this study only focused on adult gym members of 18 years and above. The study’s design was a quantitative cross-sectional design as described by Graziano and Raulin ^[13]^, using a questionnaire that allowed for multiple responses as the data were collected and validated based on a study undertaken by Gradidge ^[14]^. The sample size was computed, based on the work of Sekaran (1992)^[15]^ The sampling method used was a systematic convenience sampling method consisting of male and female gym goers who attended the gym twice or more times per week as presented in an earlier study by Mc Creanor et al.,.^[16]^ The questionnaires were personally administered by the researcher and were available to provide clarification to the participants if necessary. The process of recruitment involved a random selection, that is, by asking every third person entering the gym whether they would be willing to volunteer for the study. Refusal to participate meant the subsequent person was asked and thereafter the recruitment process reverted again to every third person. In this study, protein supplements are defined as commercially available products marketed by the manufacturers to provide for a claimed human nutritional deficiency. Natural source protein is acquired, often as whole food, of plant or animal origin. Spiritual motives relate to the feeling or belief that there is a person’s training is not only for the physical aspects derived from training, but that there is also a mental or spiritual feature that is also achieved. In the context of the consumption of nutritional supplements, the theory of attitudes, namely, the functionalist theory by the psychologist Daniel Katz was used as a guideline in this study. ^[17]^ The theory is formulated by its functions, and articulates why people hold on to certain attitudes which may assist them in attaining their basic goals and needs. ^[17]^

### Questionnaire approach and methodology

The questionnaire’s content for this study specifically covers, (i) reasons for attending a gym by means of gender, (ii) nutritional supplement consumption by gym users, (iii) age of first consumption of nutritional supplements, (iv) the monthly cost of supplements, and (v) examples of nutritional protein containing supplement products. It covers aspects not previously published by the researchers Mc Creanor, et al. related to this study. Study participants were assured of confidentiality, prior to their providing research consent. The participants’ self- administered questionnaires were numbered but remained anonymous. The questionnaires were approved by the University of Johannesburg, Faculty of Health Sciences Research Ethics Committee (REC-01-144-2015).

### Statistical analysis

Data for the study were manually transcribed and independently validated using the Windows-based Microsoft ® Office Excel 2003 SP 1 (Excel © 1985-2003 Microsoft Corporation). The Statistical Package for the Social Sciences (SPSS Inc. version 16) was used to calculate the descriptive and inferential statistics. The respective results are presented as percentages.

## Results

The age of the gym goers in the Johannesburg North area ranged between 18–49 years (mean 27 years). Of the total of 364 participants, 205 (56%) were male and 159 (44%) were female.

The main reasons for attending the gyms as presented in Table 1 (multiple responses), show that female participants in the study indicated and based on the percentage response was for muscle gain (57%), weight loss (48%), and staying healthy (47%). For the male participants in the study, the main reasons for attending the gym and based on percentage response were for muscle gain (54%), cross-fit training (33%), and body conditioning (29%). Further, a larger percentage of male participants in the study who stated, ‘sometimes’ for reasons for attending a gym indicated the need for aerobic exercise (53%), and swimming (51%).

Table 2 contains information provided by the participants on supplement use. These are protein supplements (84%), carbohydrates (72%), and vitamins (71%). Supplements that were consumed to a lesser extent by the study cohort were Phedra-Cut thermogenic aids (35%), branched-chain amino acids (BCAA) (34%), and L-glutamine (33%).

Table 3 illustrates the age of the study’s participants first use of nutritional supplements. Of the cohort (n=216), (60%) (n=130) indicated that they commenced using nutritional supplements between the ages of 13- to 18-years-old (high school level), (23%) (n=49) between the ages 10- to 12- years-old (primary school level), and (17%) (n=37) were over 18-years-old when they joined the gym.

Of the total of 362 respondents, 48% (n=174) ‘sometime’ read the label’s information, 41% (n=149) ‘always’ read the label’s information, and 11% (n=39) ‘never’ read the label’s information for nutritional value, benefits and side effects.

Table 4 illustrates the monthly spend (in South African Rands) on performance-enhancing supplements by the study’s participants.

Of the study’s participants (n=222), 26% (n=58) indicated that they spent between R500 to R1000 per month on nutritional supplements at the time of the study. Twenty-four percent (n=54) spent up to R500, 23% (n=51) spent between R1000 to R1500, 9% (n=20) spent between R2000 to R2500, 2% (n=3) spent between R2500to R3000, and 1% (2) spent than R3000 per month.

Tables 5 and Table 6 respectively provide a comparative illustration of protein-containing dietary/nutritional to that of ‘natural protein source’ products.

## Discussion

The study was focussed on assessing Johannesburg North gym goers’ reasons for going to the gym, their specific nutritional product consumption, and the monthly cost to attain their personal goals and needs. The demography of the study’s participants ranged between 18–49 years (a mean of 27 years). Of the total of 364 participants, 205 (56%) were male and 159 (44%) were female.^[16]^ The study data (Table 1) show that females were more *‘certain’* with their reasons for attending the gym, than males based on the sport/exercise type that were provided as options in the questionnaire. The main reasons (Table 1) for female participants in the study to attend the gym indicated with *‘certainty’* were for muscle gain (57%) and weight loss (48%). Male participants indicated with *‘certainty’* that their reason for attending the gym was for muscle gain (54%). This finding concurs with Senekal et al, ^[7]^ which indicated that supplement use and exercising by younger men seem to focus on muscle building/strength and fitness. ^[7, 18]^

Further, this study shows that the female participants (39%) had a ‘spiritual motive’ for attending the gym compared to males who indicated that they ‘never’ had such a purpose.

Table 2 shows that of the overall cohort of gym patrons, 84% of the participants indicated that they consume protein supplements, followed by carbohydrates (72%), and vitamins (71%). Of the supplements that were consumed to a lesser extent by the study’s cohort was PhedraCut thermogenic aids (35%), branched-chain amino acids (BCAA) (34%), and L-glutamine (33%). This study’s findings concur with those of Jonvik, et al. ^[8]^ which showed an increasing interest in the role of protein ingestion during and after endurance exercise to support physiological training adaptations ^[8]^. The use of protein supplements is an important finding for the overall cohort, and can be associated with the muscle gain requirement as shown in Table 1 for both male and female participants.

Two hundred and nineteen (60%) of the participants indicated that they started using nutritional supplements between 13–18 years (high school), 84 (23%) between 10–12 years (primary school), and 62 (17%) were over 18 years old (Table 3). These results indicate how early in life supplements were used and continued into adulthood, concurring with the findings of ^[2,7]^ relating to the prevalence of supplement use. ^[2,7]^. This shows the impact and attraction that nutritional supplements have on adolescents. Further, as in the work of Gabriels et al. ^[3]^ it shows that certain products have age restriction consumption on the product’s label, but which may be disregarded by both the consumer and the supplier through ‘aggressive’ marketing practices. ^[19]^

As published in a previous work by Mc Creanor et al. ^[17]^ the main sources of information regarding nutritional supplements were the Internet (75%), pharmacists (67%) and books (66%). The provision of information on supplements which was least accepted by the respondents was from parents (38%), siblings (37%), physicians (36%), and biokineticists (34%).

The study by van der Walt and Coopoo ^[20]^ also identified pharmacists as a main source of this information.^[20]^ Contrary to this study’s findings, the research of Gabriels and Lambert ^[19]^ showed that (43%) of their respondents where not influenced by information on nutritional supplements provided by pharmacists, dieticians, nutritionists and doctors. ^[19]^ Instead, 24% of respondents indicated that they were influenced by information provided by the coach, gym and/or fitness trainer, and fellow athletes. ^[19]^ What the various studies show is that information acquisition is changing rapidly. ^[16,20]^ This is all the more important for continuous research practice, on how information-gathering occurs to ensure the reliability of information provided in the interest of the well-being of the consumer.

Out of the total of 362 respondents, 48% (n=174) ‘sometimes’ read the label’s information, 41% (n=149) *‘*always’ read the label’s information, and 11% (n=39) ‘never’ read the label’s information for a product’s nutritional value, benefits and side effects. The study by Gabriels and Lambert ^[19]^ showed that with particular emphasis on moderately physically active and competitive participants, close to 70% of the respondents who purchased supplements were strongly influenced by label’s information, specifically that the product should to be free of banned substances and be of a high quality. ^[19]^ Whilst this may be true for the particular cohort studied, what is undeclared on the label’s content (e.g. steroids) may be the ‘driver of consumption’, knowingly or unknowingly. ^[19]^ This ‘driver of consumption’ will assist in attaining the gym user’s personal goals and needs, which is in line with the functionalist theory of psychologist Daniel Katz. ^[17]^

Table 4 illustrates the monthly costs at the time of the study (in South African Rands) on performance-enhancing supplements by study participants who attended the Johannesburg North gym. Of the study’s participants (n=222), 26% (n=58) indicated that they spent between R500-R1000 per month on nutritional supplements at the time of the study. Twenty-four percent spent up to R500, 23% spent between R1000-R1500, 9% spent between R2000-R2500, 2% spent between R2500-R3000, and 1% spent more than R3000. Thus a sizable proportion based on the first three categories (Table 4), account for 73% of the study participants who spent about R1500 or less per month on nutritional supplements.

Recent evidence suggests that the protein requirements of well-trained endurance athletes, performing at high levels of endurance exercise training may be greater than previously assumed. ^[8,10]^

Gym users see people with their desired body image and often, without considering the consequences, go to great lengths to achieve these goals. The fear of not being able to achieve the goals contributes to changing their behaviour towards the use of performance-enhancing supplements, even though they are opposed to the idea of taking these supplements. ^[17]^ Hence, many gym users in this study support this growing market. This is notwithstanding that this behaviour may steer gym users in the wrong direction, as taking these supplements may not necessarily be best for their health and well-being. ^[17]^

Table 5 and Table 6 illustrate the use of protein-containing nutritional and natural source products. Natural source protein-containing products, such as chicken, peanut butter, and beans (Table 6) are cheaper per 100 grams of protein than the nutritional supplement products (Table 5). The work of Nowson and O’Connell ^[21]^ indicates that for a subject weighing 72 kg, the Recommended Daily Allowance (RDA) for protein is 58–86 grams per day. ^[21]^ To maximise muscle protein accretion with resistance exercise, the daily protein intake should be approximately between 115–158 grams In order to retain lean body mass during weight loss protein intakes of 167–223 grams have been advocated. ^[21]^ Based on these respective categories and levels of activity, protein recommendation could be attained cost-effectively (Table 6) via non-nutritional supplement products. Nowson and O’Connell ^[21]^ states that additional protein intake is unnecessary if a balanced diet is followed. ^[21]^ On the contrary, excessive consumption of protein can produce toxic effects that may lead to health problems, such as kidney disease, water retention and high levels of ketones in the body. This reinforces the work of Senekal et al ^[7]^ that comment on the wide use of supplements, especially protein, emphasising the need for dissemination of consistent, evidence-based information on supplement recommendations and risks. ^[7]^

Regarding a shift of protein source from supplements to non-supplement sources, there can be a change in behaviour according to Katz ^[17]^, when it no longer assists its function and the individual feels unfulfilled. ^[17]^ Gym users may thus change their attitude towards nutritional supplements, if they are aware of the health risks involved in consuming these supplements. ^[2,17]^

The approach that is suggested for behaviour change is not so much as a result of the change in the information provided but rather by altering a person’s primary motivational and personality needs.^[17]^ Body image or dysmorphia appears to be a major motivating factor for taking nutritional supplements for the non-sporting gym goer.

### Study limitations

The main limitation in all similar studies is poor memory and truthfulness from participants. In the context of this study, there may be possible under/over estimation of the actual amounts spent on nutritional supplements as provided by the study participants. The estimate of protein content in respective products is based on the information assessed from the different sources as presented in Table 5 and Table 6.

## Conclusion

The study of Johannesburg North’s gym goers has shown that the gender-specific reasons for attending the gym were for females, muscle gain, weight loss, and staying healthy; while for males it was muscle gain. A further finding was the ‘spiritual motive’ for attending the gym for females, compared to that of males who did not declare such a motive. Protein supplements were the most popular of supplements consumed by the participants. The study further shows that a sizeable proportion of participants commenced consumption of nutritional supplements at high school age, with an increasing number starting at primary school level. The main sources of information on nutritional supplements were the Internet, pharmacists and books. Only forty-one percent of the participants always read the label information. The monthly amount spent on nutritional supplements accounted for by seventy-three percent of participants about R1500 or less. This study’s findings further show that natural whole food sources of protein-containing products are cheaper than other nutritional supplements. It is important to do a cost- benefit risk assessment that is practical for the consumer. It is also necessary to check whether any undesirable contaminants are present in the supplement products.

It is important to educate gym users, the general public, and the guardians of minors regarding nutritional supplement consumption and their effects.

### Recommendations

Statutory bodies, such as the South African Drug-Free Sport (SAIDS), should embark and improve on education and awareness campaigns related to nutritional supplements, based on early age of first consumption as observed in this study. Relevant subject matter on the potential harms of nutritional supplement consumption should be included in the school curriculum. Improvement of enforcement capacity with respect to the violation of a label provision by manufacturers, distributors and retailers, needs to be accelerated, with concomitant media exposure. Gyms should offer educational classes to their patrons that focus on non-supplement-based alternative protein sources, which provide a reduced risk of contamination.

### Future research

The findings in this study show that female participants had a ‘spiritual motive’ for attending the gym compared to males. Thus exploring via self-administered questionnaire(s) in detail and depth, the link related to this ‘spiritual motive’ and the consumption or non-consumption of nutritional supplements would be useful. In order to get improved information and data on nutritional supplement consumption and the monthly costs, it would be important to gather this information at the point of sale of retail outlets, and make it available to the gym user. Knowledge of multiple nutritional consumption/combination categories, as well as gender-specific consumption would expand the relevance of the information from this study. The best approaches to provide knowledge and education pertaining to all aspects of nutritional supplements to younger age groups needs to be determined, as has been observed in this study.

## Figures and Tables

**Table 1:** Reasons for attending gymnasium by gender (n= 364)

	Male n=205 (56%)			Female n=159 (44%)		
Sport/Exercise Type	Mainly (%)	Sometimes (%)	Never (%)	Mainly (%)	Sometimes (%)	Never (%)
**Muscle gain**	54	32	14	57	17	26
**Weight loss**	25	50	25	48	32	20
**Swimming**	24	51	25	39	41	20
**Body conditioning**	29	50	21	43	35	22
**Aerobic exercise**	17	53	30	44	34	22
**Specific training**	25	44	32	40	36	24
**Spiritual motive**	24	37	39	39	36	25
**Staying healthy**	28	40	32	47	36	17
**Cross-fit training**	33	39	28	40	32	28

% indicates percentage of respondents.

**Table 2:** Nutritional supplement consumption by gym users (n = 364)

Supplement	Yes (%)	No (%)
Protein supplements (e.g. Whey protein)	84	16
Carbohydrate supplements (e.g. Energade, Powerade)	72	28
Vitamins (A, B, B12, C, D and E)	71	29
Creatine supplements (e.g. Creatine Monohydrate)	67	33
Caffeine (e.g. Redbull, Guarana)	59	41
Fish oil (e.g. Omega 3 tablets)	37	63
CLA (Conjugated linoleic acid) weight loss supplements	36	64
PhedraCut Thermogenic aid	35	65
Branched-chain amino acids (BCAA)	34	66
L-glutamine	33	67

% indicates percentage of respondents.

**Table 3:** Age of first consumption of dietary/nutritional supplements (n = 216)

Age (years)	Institution	Participants (n)	Participants (%)
10–12	Primary school	49	23
13–18	High school	130	60
Over 18	When joining gymnasium	37	17

**Table 4:** Monthly cost on supplements (n=222)

Category of spending (Rand)	Participants (n)	Participants (%)
0 – 500	54	24
501–1000	58	26
1001–1500	51	23
1501–2000	34	15
2001– 2500	20	9
2501–3000	3	2
>3001	2	1

**Table 5:** Examples of nutritional protein containing supplement products and cost

Retail Pharmacy Protein Source	Claimed Estimate % of protein in product **	Cost per 100 gram of product (Rand) *	Estimate cost per 100 gram of protein (Rand)
Biogen Pro Series Bites (Code:176972000EA)	27	71	265
Real Thing Pro Protein (Code: 060822000EA)	95	149	157
USN Pure Protein Nutritional Bars (Code: 154116003EA)	27	40	147
USN Trust Protein Nutritional Bars (Code: 187675001EA)	38	50	131
NPL 100% Whey Protein Isolate Supplement (Code: 190883001EA)	83	53	64.
Evox 100% Casein Protein Supplement (Code: 082011000EA)	90	49	54
NPL Whey Protein Supplement (Code: 176978001EA)	67	32	48
Evox Synergy Whey Protein Supplement (Code: 153794006EA)	77	30	39
Primal Nutrition Whey Protein (Code: 187183001EA)	65	24	37
Evox Synergy Whey Protein Cookies (Code: 066133000EA)	77	27	35
Future Life High Protein (Code: 167580001EA)	30	10	32
Biogen Pro Series Nitro Protein Supplement (Code: 105586000EA)	77	32	25

* Cost as assessed in February 2018 retail pharmacy (Dischem) https://www.dischem.co.za/search/?q=protein+supplements. Accessed 24th July 2019.

** https://www.predatornutrition.com/articlesdetail?cid=9-cheap-sources-of-protein

**Table 6:** Example of ‘natural sources’ of protein containing products (alternative) and cost

Retail Market Protein Source	Claimed Estimate % of protein in product	Cost per 100 gram of product (Rand) *	Estimate cost per 100 gram of protein (Rand)
Tofu	8	14	170
Nuts	20	30	150
Hemp powder	45	61	136
Sardines	25	21	84
Beef	26	13	51
Milk	3.4	2	44
Eggs	13	4	34
Chicken	31	6	19
Peanut butter	25	5	19
Beans	21	4	17

* Cost as assessed in October 2018 retail market (Pick ‘n Pay)
